# Whole-Brain Neural Connectivity to Lateral Pontine Tegmentum GABAergic Neurons in Mice

**DOI:** 10.3389/fnins.2019.00375

**Published:** 2019-04-24

**Authors:** Ze-Ka Chen, Xiang-Shan Yuan, Hui Dong, Yong-Fang Wu, Gui-Hai Chen, Miao He, Wei-Min Qu, Zhi-Li Huang

**Affiliations:** ^1^State Key Laboratory of Medical Neurobiology and MOE Frontiers Center for Brain Science, Institutes of Brain Science, Department of Pharmacology, School of Basic Medical Sciences, Fudan University, Shanghai, China; ^2^Department of Neurology (Sleep Disorders), Chaohu Hospital of Anhui Medical University, Hefei, China

**Keywords:** GABA, lateral pontine tegmentum, viral neuronal tracing, reciprocal connections, sleep-wake cycle, locomotion

## Abstract

The GABAergic neurons in the lateral pontine tegmentum (LPT) play key roles in the regulation of sleep and locomotion. The dysfunction of the LPT is related to neurological disorders such as rapid eye movement sleep behavior disorder and ocular flutter. However, the whole-brain neural connectivity to LPT GABAergic neurons remains poorly understood. Using virus-based, cell-type-specific, retrograde and anterograde tracing systems, we mapped the monosynaptic inputs and axonal projections of LPT GABAergic neurons in mice. We found that LPT GABAergic neurons received inputs mainly from the superior colliculus, substantia nigra pars reticulata, dorsal raphe nucleus (DR), lateral hypothalamic area (LHA), parasubthalamic nucleus, and periaqueductal gray (PAG), as well as the limbic system (e.g., central nucleus of the amygdala). Further immunofluorescence assays revealed that the inputs to LPT GABAergic neurons were colocalized with several markers associated with important neural functions, especially the sleep-wake cycle. Moreover, numerous LPT GABAergic neuronal varicosities were observed in the medial and midline part of the thalamus, the LHA, PAG, DR, and parabrachial nuclei. Interestingly, LPT GABAergic neurons formed reciprocal connections with areas related to sleep-wake and motor control, including the LHA, PAG, DR, parabrachial nuclei, and superior colliculus, only the LPT-DR connections were in an equally bidirectional manner. These results provide a structural framework to understand the underlying neural mechanisms of rapid eye movement sleep behavior disorder and disorders of saccades.

## Introduction

The pontine tegmentum, a brainstem structure, are all the regions from the basilar pons to the fourth ventricle. It plays important roles in perception, movement, vigilance, respiration, and the sleep-wake regulation (Plazzi et al., 1996; Alheid et al., 2004; Tziridis et al., 2012). The lateral pontine tegmentum (LPT) is the dorsolateral part of the pontine tegmentum, which is included in the deep mesencephalic nucleus (DpMe). The LPT is identified as the site between the ventral lateral periaqueductal gray (vlPAG) and the pedunculopontine tegmental nucleus (PPT) on the ventro-dorsal localization, and is a trip lying the region medial to the PPT on the rostro-caudal extent in rodents (approximately -4.04 to -5.02 mm corresponding to Bregma in the mouse brain atlas) (Franklin and Paxinos, 2001; Lu et al., 2006; Sherman et al., 2015). The LPT mainly contains glutamatergic and GABAergic neurons (Liang et al., 2014).

The LPT is involved in voluntary locomotion, general anesthesia, and even incentive learning (Sukhotinsky et al., 2005; Fogel et al., 2010; Le Ray et al., 2011). Dysfunction of the LPT is associated with neurological disorders such as rapid eye movement (REM) sleep behavior disorder (RBD), saccade disorders and temporal lobe epilepsy (TLE). Using magnetic resonance imaging, diffusion changes of visualized structural abnormalities were found in the areas including the LPT in patients with idiopathic RBD (Scherfler et al., 2011). Ocular flutter was found to be related to the right upper pontine tegmentum damage, which contained LPT neurons (Tsutsumi et al., 2009). LPT dysfunction in patients with TLE was found to contribute to neurocognitive disturbances (Englot et al., 2018). In addition to the above, LPT neurons are especially known to have a vital function in REM sleep regulation in rodents (Sapin et al., 2009) and demyelination of LPT neurons led to dream-like hallucinations during wake, relating to the impairment of REM sleep inhibitory mechanisms (Vita et al., 2008). Furthermore, c-fos, a marker of activated neurons, was strongly expressed in the LPT after REM sleep deprivation and colocalized with glutamate decarboxylase, a key enzyme for the production of GABA, suggesting that LPT GABAergic neurons are involved in REM sleep regulation (Lu et al., 2006; Sapin et al., 2009).

The neuronal connectivity of LPT neurons has previously been investigated using classic anterograde tracers, such as biotinylated dextran amine (BDA), and retrograde tracers, such as horseradish peroxidase or cholera toxin b-subunit (CTB). Consequently, the LPT was shown to receive inputs from the lateral hypothalamic area (LHA) (Clement et al., 2012), the pallidum (Gorbachevskaia, 2011), and the sublaterodorsal nucleus (SLD) (Boissard et al., 2003), while projecting to the LHA (Ford et al., 1995), superior colliculus (SC) (Appell and Behan, 1990), and SLD (Liang and Marks, 2014). However, specific afferent inputs and efferent outputs of LPT GABAergic neurons remain unknown.

Recent genetic tracing methods using the retrograde trans-synaptic rabies virus (RV) allow us to identify the presynaptic connections of a genetically well-defined neuronal population (Wickersham et al., 2007; Yuan et al., 2018). Moreover, the use of adeno-associated virus (AAV) vectors allows cell-type-specific, non-toxic, long-term expression of transgenes for anterograde tracing of neuronal pathways (Kuhlman and Huang, 2008; Holloway et al., 2013; Zhang et al., 2013). Here, we employed a modified RV and AAV in combination with the Cre/LoxP system to map presynaptic inputs and output projections of LPT GABAergic neurons. We identified several monosynaptic inputs of LPT GABAergic neurons by double labeling with antibodies for molecular markers related to sleep-wake regulation. Our results revealed the wide-ranging connectivity of LPT GABAergic neurons and provided a structural framework to understand the underlying neural circuits of important physiological functions.

## Materials and Methods

### Animals

The viral-based tracing experiments were all performed in male GAD2-IRES-Cre mice obtained from Miao He and Z. Josh Huang’s laboratory, which generated the strain on a mixed genetic background (129SVj/B6; with black coat color) to genetically target almost whole-brain GABAergic neurons with a high level of specificity (Taniguchi et al., 2011). The mice were bred and housed under an automatically controlled 12-h light/12-h dark cycle (lights on at 7 a.m.; 100 lux intensity) (Zhang et al., 2017). After surgery, the mice were allowed to recover for at least 2 weeks before further experiments. This study was carried out in accordance with the recommendations of the China Regulations on the Administration of Laboratory Animals (the Decree No. 2 of National Science and Technology Commission of the People’s Republic of China) and all animal procedures were approved by the Medical Experimental Animal Administrative Committee of the School of Basic Medical Sciences (Permit No. 20140226-024), Fudan University (Shanghai, China).

### Virus

All the viral vectors were packaged by BrainVTA (BrainVTA, Co., Ltd., Wuhan, China). The titer of the EnvA-pseudotyped, glycoprotein (RG)-deleted, DsRed-expressing rabies virus (RV-EnvA-ΔRG-DsRed) was about 2 × 10^8^ infecting units per mL. The three AAV vectors, namely AAV-EF1α-DIO-ChR2-mCherry, AAV-EF1α-DIO-TVA-GFP, and AAV-EF1α-DIO-RV-G, were all packaged into the 2/9 AAV serotype and titred at about 3 × 10^12^ genome copies per mL (Yuan et al., 2018).

### Surgery and Viral Injections

Totally, 28 mice were injected with viruses. Only eight animals were correctly injected after histological confirmation and were included in the results. For virus injection, adult GAD2-IRES-Cre mice were anesthetized with ∼1.5% isoflurane in oxygen (flow rate of 1 L/min). Using the Nanoject II (Drummond Scientific, Broomall, PA), 23 nL AAV9-EF1α-DIO-TVA-GFP and AAV9-EF1α-DIO-RvG was stereotaxically injected (4.6 nL/s) via a micropipette into the unilateral LPT (anterior–posterior: -4.2 mm, medial-lateral: +1.0 mm, dorsal-ventral: -3.5 mm) (Krenzer et al., 2011). Following the injection, the pipette was held in place for an additional 10 min to allow diffusion of viral particles away from the injection site before being slowly withdrawn. Two weeks later, 46 nL volume of RV-EnvA-ΔRG-DsRed was prepared to inject into the previous site of the LPT (*n* = 4 mice). For anterograde tracing, AAV-EF1α-FLEX-ChR2-mCherry was injected into the LPT following the same procedures described above. After 3 weeks, these mice were perfused (*n* = 4 mice).

### Histology and Immunostaining

After being deeply anesthetized, adult mice were transcardially perfused with cold normal saline followed by 4% paraformaldehyde in 0.1 M phosphate buffer (PB). The brains were then extracted, post-fixed for 6 h, and then incubated in 20% sucrose in PB at 4°C until they sank. Coronal sections (30 μm) were cut from a fixed brain on a freezing microtome (CM1950, Leica, Germany) into four series to obtain groups of tissue for multiple manipulations, and the distance between sections in each group was 90 μm. Tissue groups were restored in a cryopreservative solution.

For immunofluorescence, sections were rinsed with 0.3% Triton X-100 in 0.01 M PBS, and then they were incubated by primary antibodies [goat anti-choline acetyltransferase (ChAT), 1: 1000, Millipore AB144P; polyclonal rabbit anti-GABA, 1:1000, Acris Antibodies 20094; polyclonal rabbit anti-5-hydroxytryptamine (5-HT), 1:3000, Sigma S5545; polyclonal rabbit anti-calretinin, 1:2000, Invitrogen MA5-14540; polyclonal goat anti-parvalbumin, 1:3000, Swant PVG213; polyclonal goat antibody against orexin A, SCB sc8070, 1:2000; polyclonal goat antibody against melanin-concentrating hormone (MCH) sc-14509, SCB,1:1000] in PBS containing 0.3% Triton X-100 (PBST) overnight at 4°C. Then the sections were washed and incubated by secondary antibodies (AlexaFluor 488 donkey antibody against goat IgG, 1: 500, Jacksonimmuno, Inc.; AlexaFluor 488 donkey antibody against rabbit IgG, 1: 1000, Jacksonimmuno, Inc.; AlexaFluor 647 donkey antibody against goat IgG, 1: 1000, Jacksonimmuno, Inc.) for 1 h at room temperature. Next, the sections were incubated for 10 min to stain nuclei by 4′,6-diamidino-2-phenylindole (DAPI, 1: 10000, Sigma-Aldrich D9542) and rinse washed three times. Finally, the sections were mounted on glass slides and cover-slipped. For the immunostaining of mCherry, free-floating sections were rinsed in PBS and incubated by primary antibodies (polyclonal rabbit anti-mCherry 1:3000, Clontech 632496) in PBST and waggled slowly overnight at 4°C. After washing, sections were incubated with secondary antibodies (biotinylated goat anti-rabbit IgG, 1:1000, Vector Laboratories BA-1000) and then incubated with an avidin-biotin peroxidase complex (ABC) solution (1:1000, Vector Laboratories PK-6100) for 1 h. After washing, the sections were immersed in a 3,3-diaminobenzidine-4 HCl (DAB, Vector Laboratories SK-4100) for 5–10 min at room temperature, after which mCherry-immunoreactive neurons could be identified by the presence of brown reaction product. Finally, the sections were mounted on glass slides, dried, dehydrated, and cover-slipped (Chen et al., 2016; Yuan et al., 2017; Luo et al., 2018).

### Imaging and Analysis

Whole-brain sections were imaged by a 10 or 20× objective on the VS120 virtual microscopy slide scanning system (Olympus) and magnified images of brain sections were captured using a 20 or 40× objective by a confocal microscope, to obtain more detail (Olympus Fluoview 1000, Tokyo, Japan).

For cell mapping of neurons, neural bodies were quantified semi-automatically using ImageJ software. Based on mouse brain map, ImageJ was used to manually depict the boundaries of specific brain regions (Franklin and Paxinos, 2001). For starter cell mapping, we first applied ImageJ to distinguish the cells co-expressing DsRed and GFP as starter cells, then generated cell representation by applying the automatic wand (tracing) tool and bicubic interpolation to maximize neuronal fidelity. Next, we inverted the colorless areas to white and matched the cellular outlines to the appropriate brain regions based on the mouse brain atlas (Figure 2A). Starter cells were binned at 0.12 mm along the anterior–posterior axis, centered at the brain slice coordinate for each coronal section image.

For axonal varicosity counting, the images were captured by a 20× objective on the Olympus VS120 system. The axonal varicosity values of overall brain were calculated semi-automatically by particle analyzing plugin in ImageJ. If the axons had a transverse diameter greater than 0.5 μm, then varicosities were defined (Mechawar et al., 2000; Li et al., 2002). The outlines of brain regions were also depicted by ImageJ based on the reference brain atlas.

All data values were presented as the mean ± standard error of the mean (SEM). The strength and direction of the linear relationship between subregions and cells or varicosity proportion were measured by the Pearson product-moment correlation coefficient (CC). Two-tailed Student’s *t-*tests were used to compare the monosynaptic inputs and axonal projections of LPT GABAergic neurons.

## Results

### Applying the Rabies Virus System to Identify Monosynaptic Inputs to LPT GABAergic Neurons

**FIGURE 1:**
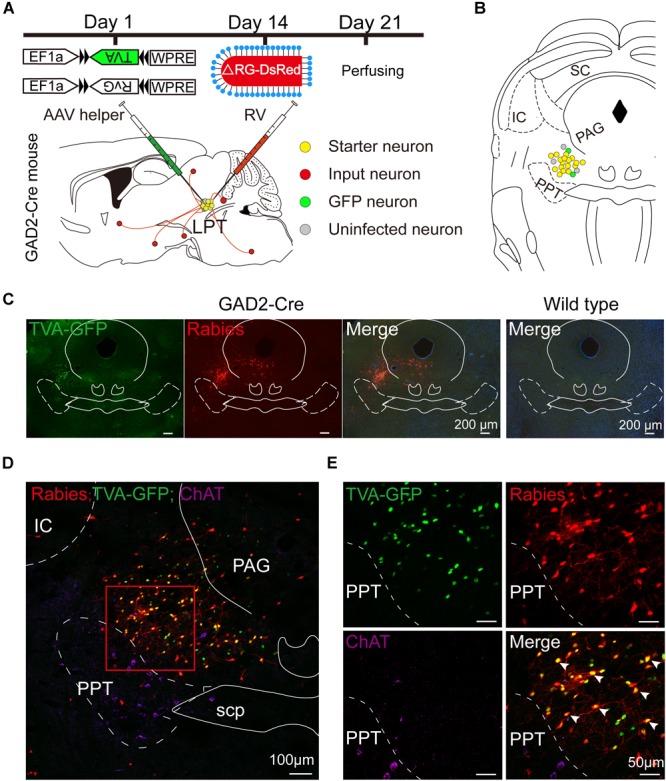
Experimental strategy for rabies-based retrograde tracing in LPT GABAergic neurons. **(A)** A schematic of the viral vectors and injection procedure for helper virus (AAV-EF1α-DIO-TVA-GFP and AAV-EF1α-DIO-RvG) and RV-EnvA-ΔRG-DsRed into the LPT of GAD2-Cre mice. **(B)** A schematic showing a coronal view of the anatomical localization and virus infection in the LPT. **(C)** Representative images showing GFP- and DsRed-expressing neurons in the LPT after helper virus and rabies virus injections in a GAD2-IRES-Cre mouse but not in a wild-type mouse. **(D)** Fluorescence images showing that the viruses were restricted to the unilateral LPT between the pedunculopontine tegmental nucleus (PPT, labeled in green by ChAT) and the periaqueductal gray (PAG) in a GAD2-IRES-Cre mouse. **(E)** An enlarged view of the red boxed region in **(D)** shows that starter cells (marked by arrowheads) co-expressed GFP with DsRed and not overlapped with ChAT immunoreactive signals.

In order to identify monosynaptic inputs to LPT GABAergic neurons, we used a cell-type-specific, RG-deleted RV strategy in GAD2-IRES-Cre mice. The approach has been reported to mark monosynaptic inputs to specifically selected starter cells and has been successfully used in previous studies by our group (Yuan et al., 2018). GAD2-IRES-Cre mice were injected in the LPT with AAV helper viruses fused to green fluorescent protein (GFP). Two weeks later, the modified RV was injected in the same area. One week after this second injection, the mice were perfused, and the brains were processed (Figure 1A,B).

**FIGURE 2:**
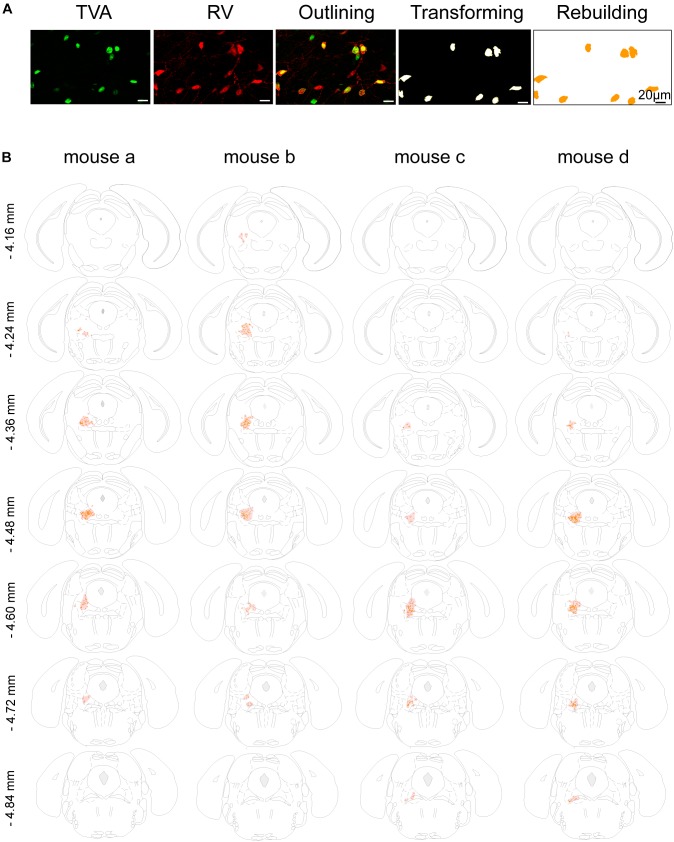
Mapping of the starter cells in the LPT. **(A)** An image series showing the rebuilding procedure of the starter cells. **(B)** Coronal sections showing the distribution of starter cells in the LPT (between –4.16 and –4.84 mm from bregma) of four GAD2-IRES-Cre mice. The images were reconstructed in ImageJ to identify brain regions based on the reference brain atlas; starter cells are indicated by the orange points and the shaded region.

Rabies virus infection was clearly detectable at the injection site in GAD2-IRES-Cre mice compared with wild-type littermates, indicating no leakage of viral infection (Figure 1C). The location of the injection site was verified by identifying virus-infected neurons in the dorsomedial corner of the PPT and the ventrolateral corner of the PAG. The yellow staining in neurons indicated that the starter cells were co-infected by AAV helper viruses and RV, and the purple staining in neurons presented ChAT immunoreactive signals for the PPT, which was the border of the LPT (Figure 1D,E). There were also some DsRed-positive neurons not expressing GFP in the LPT, revealing the existence of local input directly to LPT GABAergic neurons (Figure 1D,E, 2A).

In addition, we mapped the GABAergic starter cells as described in Section “Materials and Methods” (Figure 2A) and revealed that they were mostly restricted to the LPT. Most of the starter neurons were found to be located in the coronal planes between -4.16 and -4.84 mm from bregma, especially between -4.36 and -4.60 mm from bregma (Figure 2B).

### Input Patterns of LPT GABAergic Neurons

**FIGURE 3:**
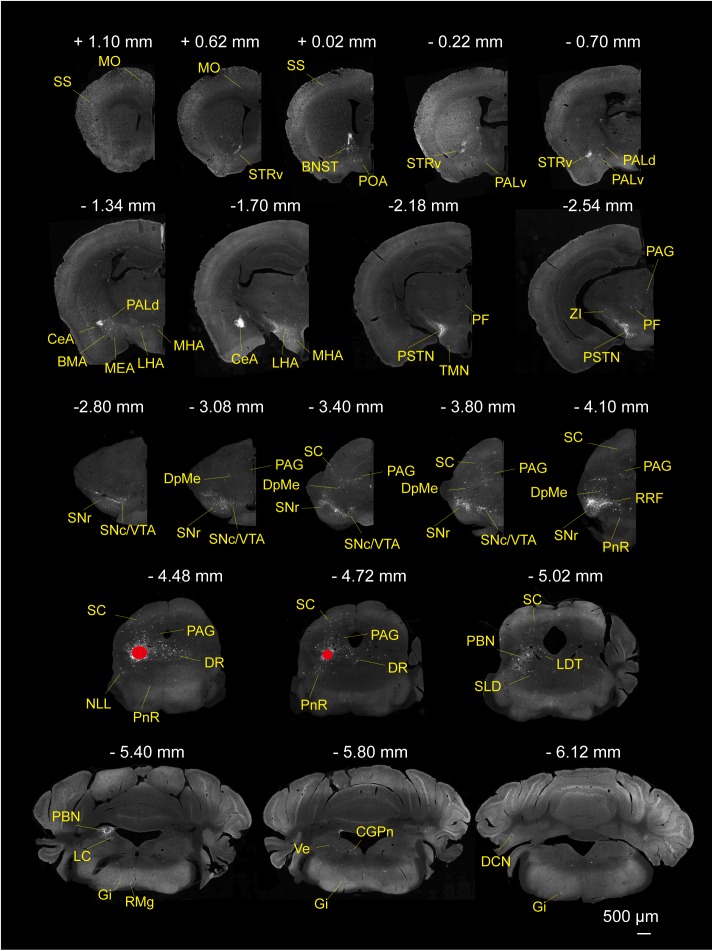
Monosynaptic inputs to LPT GABAergic neurons. Representative coronal sections showing the monosynaptic inputs to LPT GABAergic neurons. The injection site is marked by red solid circles. Only the hemisphere ipsilateral to the injection area is shown. Scale bar: 500 μm. MO, momatomotor cortex; SS, somatosensory cortex; STRv, stratum ventral region; PALv, pallidum, ventral region; PALd, pallidum, dorsal region; BMA, basomedial amygdalar nucleus;CeA, central amygdaloid nucleus; MEA, medial amygdalar nucleus; BNST, bed nucleus of the stria terminalis; PF, parafascicular thalamic nucleus; POA, preoptic area; LHA, lateral hypothalamic area; MHA, medial hypothalamic area; PSTN, parasubthalamic nucleus; TMN, tuberomammillary nucleus; ZI, zona incerta; SNc, substantia nigra pars compacta; SNr, substantia nigra pars reticulata; DpMe, deep mesencephalic nucleus; VTA, ventral tegmental area; SC, superior colliculus; RRF, retrorubral field; DR, dorsal raphe nucleus; PAG, periaqueductal gray; PnR, pontine reticular nucleus; NLL, the nuclei of the lateral lemniscus; PBN, parabrachial nucleus; SLD, sublateral dorsal nucleus; LDT, laterodorsal tegmental nucleus; LC, locus coeruleus; Gi, gigantocellular reticular nucleus; CGPn, central gray of the pons; RMg, raphe magnus nucleus; Ve, vestibular nucleus; DCN, deep cerebellar nucleus.

**FIGURE 4:**
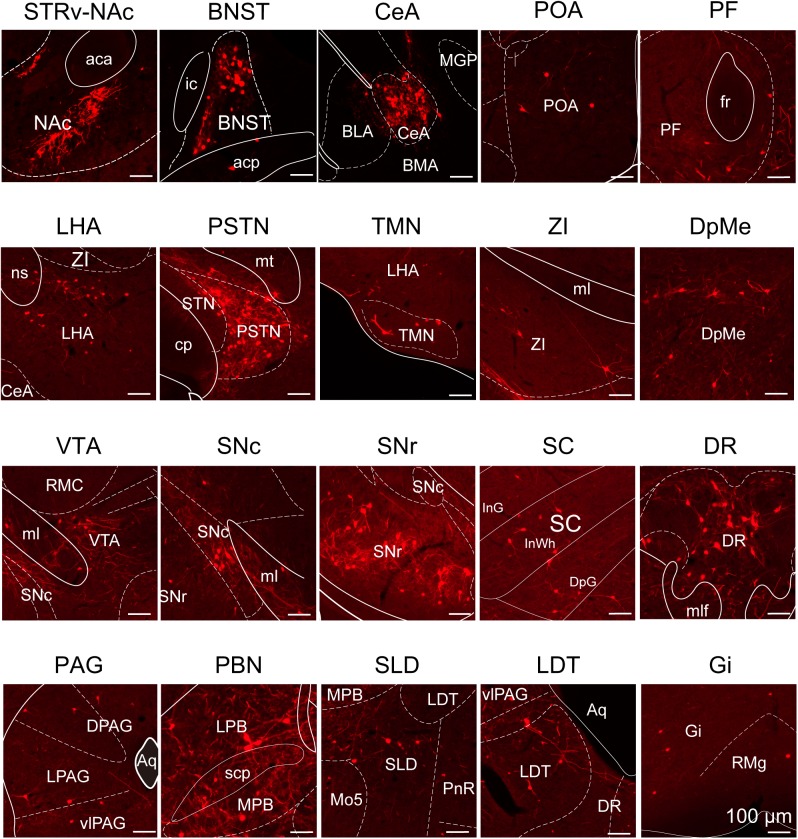
Schematic presenting the typical regions with monosynaptic inputs to LPT GABAergic neurons. Primary inputs originated from regions involved in sleep-wake regulation (e.g., LHA, TMN, and SLD), locomotion (e.g., SC and SNr), emotion (e.g., CeA, and DR), and reward control (e.g., VTA and NAc). Scale bar: 100 μm. STRv, stratum ventral region; NAc, nucleus accumbens; BNST, bed nucleus of the stria terminalis; POA, preoptic area; CeA, central amygdaloid nucleus; BLA, basolateral amygdalar nucleus; BMA, basomedial amygdalar nucleus; MGP, medial globus pallidus; ZI, zona incerta; STN, subthalamic nucleus; PSTN, parasubthalamic nucleus; LHA, lateral hypothalamic area; PF, parafascicular thalamic nucleus; TMN, tuberomammillary nucleus; RMC, red nucleus; SNc, substantia nigra pars compacta; VTA, ventral tegmental area; SNr, substantia nigra pars reticulata; PAG, periaqueductal gray; dPAG, dorsal periaqueductal gray; lPAG, lateral periaqueductal gray; vlPAG, ventrolateral periaqueductal gray; SC, superior colliculus; PnR, pontine reticular nuclues; DR, dorsal raphe nucleus; PBN, parabrachial nucleus; LPB, lateral parabrachial nucleus; MPB, medial parabrachial nucleus; SLD, sublateral dorsal nucleus; LDT, laterodorsal tegmental nucleus; Mo5, motor trigeminal nucleus; RMg, raphe magnus nucleus; Gi, gigantocellular reticular nucleus; aca, anterior commissure, anterior part; ic, internal capsule; fr, fasciculus retroflexus; ns, nigrostriatal bundle; cp, cerebral peduncle, basal part; mt, mammillothalamic tract; ml, medial lemniscus; InG, intermediate gray layer of the superior colliculus; InWh, intermediate white layer of the superior colliculus; DpG,; mlf, medial longitudinal fasciculus; Aq, aqueduct; scp, superior cerebellar peduncle.

**FIGURE 5:**
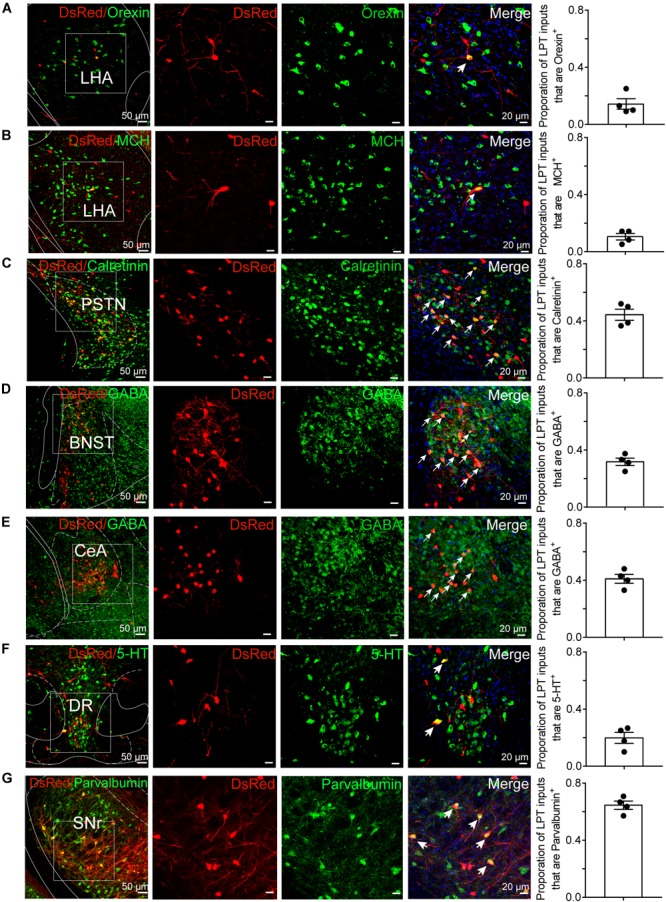
Immunofluorescence of DsRed and several markers of sleep-wake regulation. **(A–E)** Images showing DsRed-labeled afferent neurons and several markers of sleep-wake regulation in typical brain regions. Enlarged views of the white boxed regions in the leftmost columns are shown in the three right columns. Colocalized neurons are indicated by arrows in the merged images (×40 magnification). Images displaying that some DsRed-labeled neurons colocalized with orexin^+^, MCH^+^, or calretinin^+^ neurons in the hypothalamus **(A–C)**, GABAergic neurons in the BNST or CeA **(D,E)**, 5-HT-positive neurons in the DR **(F)**, and parvalbumin (PV)-positive neurons in the SNr **(G)**. Rightmost columns, quantification of DsRed+ cells that are positive for special biomarkers. *n* = 4, each data point represents one experimental animal. LHA, lateral hypothalamic area; PSTN, parasubthalamic nucleus; CeA, central amygdaloid nucleus; DR, dorsal raphe nucleus; SNr, substantia nigra pars reticulata; MCH, melanin-concentrating-hormone; 5-HT, 5-hydroxytryptamine.

To determine the presynaptic connections of LPT GABAergic neurons, we analyzed serial coronal sections from mice injected with TVA-based trans-synaptic tracer. Sections from a representative GAD2-IRES-Cre brain revealed RV-DsRed-labeled presynaptic input neurons located only in specific brain nuclei in a unilateral manner (Figure 3). Figure 4 depicts input tracing to LPT GABA neurons in greater detail, with enlarged images of inputs from typical subregions such as the bed nucleus of the stria terminalis (BNST), central nucleus of the amygdala (CeA), parasubthalamic nucleus (PSTN), substantia nigra pars reticulata (SNr), SC, dorsal raphe nucleus (DR), PAG, and tuberomammillary nucleus (TMN). Further immunofluorescence assays showed that the inputs to LPT GABAergic neurons were colocalized with several markers associated with important neural functions, especially the sleep-wake cycle. In the hypothalamus, the inputs to LPT GABAergic neurons were found to be partly colocalized with orexin (0.14 ± 0.04 in Figure 5A) or MCH (0.11 ± 0.02 in Figure 5B) in the LHA, which was shown to inhibit or promote REM sleep (Adamantidis et al., 2007; Sasaki et al., 2011; Vetrivelan et al., 2016). The input neurons from the PSTN to LPT GABAergic neurons were mostly colocalized with calretinin (CR, 0.44 ± 0.04 in Figure 5C), where the inputs might to be related to the sleep-wake cycle (Steininger et al., 1999). In addition, the inputs from the limbic system to the LPT were partly colocalized with GABA neurons, such as the BNST (0.32 ± 0.03 in Figure 5D) and CeA (0.41 ± 0.03 in Figure 5E), which may be associated with sleep-wake regulation (Kodani et al., 2017; Mahoney et al., 2017) The inputs from the DR to the LPT were found partly colocalized with serotonin (0.20 ± 0.04 in Figure 5F), which was proved to promote wakefulness (Ito et al., 2013). Beyond our expectation, numerous inputs from the LPT were colocalized with parvalbumin in the SNr (0.65 ± 0.03 in Figure 5G), a region that was reported to mostly contain GABAergic neurons and to be possibly involved in REM sleep (Pal and Mallick, 2009).

**FIGURE 6:**
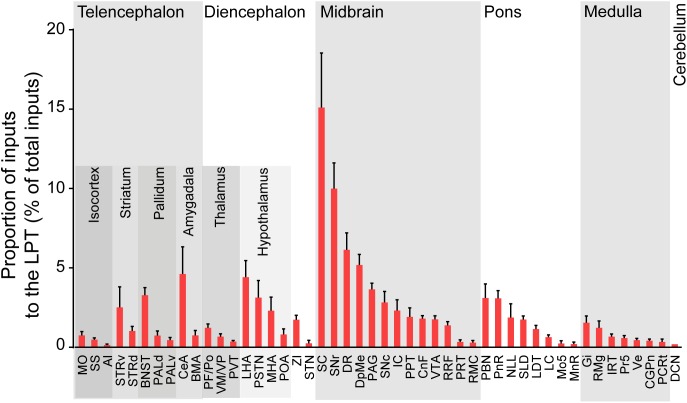
Statistical analysis of whole-brain, monosynaptic inputs to LPT GABAergic neurons. Average proportion of cell number in each brain region with more than 0.1% average input proportions from LPT GABAergic neurons. Error bars represent the SEM. Brain regions are grouped into six general structures as listed at the top and specific brain regions are listed at the bottom. MO, momatomotor cortex; SS, somatosensory cortex; AI, agranular insular cortex; STRv, stratum ventral region; STRd, striatum dorsal region; CeA, central amygdaloid nucleus; BMA, basomedial amygdalar nucleus; BNST, bed nucleus of the stria terminalis; PALv, pallidum, ventral region; PALd, pallidum, dorsal region; PVT, paraventricular thalamic nucleus; PF/Po, parafascicular/posterior thalamic nucleus; VM/VP, ventromedial/ventroposterior thalamic nucleus; LHA, lateral hypothalamic area; MHA, medial hypothalamic area; PSTN, parasubthalamic nucleus; POA, preoptic area; ZI, zona incerta; STN, subthalamic nucleus; SNc, substantia nigra pars compacta; VTA, ventral tegmental area; SNr, substantia nigra pars reticulata; DpMe, deep mesencephalic nucleus; RRF, retrorubral field; RMC, red nucleus; CnF, cuneiform nucleus; PPT, pedunculopontine tegmental nucleus; IC, inferior colliculus; PRT, pretectal region; SC, superior colliculus; DR, dorsal raphe nucleus; PAG, periaqueductal gray; PnR, pontine reticular nuclues; NLL, the nuclei of the lateral lemniscus; LDT, laterodorsal tegmental nucleus; PBN, parabrachial nucleus; SLD, sublateral dorsal nucleus; LC, locus coeruleus; Mo5, motor trigeminal nucleus; MnR, median raphe nucleus; Gi, gigantocellular reticular nucleus; Ve, vestibular nucleus; PCRt, parvicellular reticular nucleus, alpha part; Pr5, principal sensory trigeminal nucleus; CGPn, central gray of the pons; RMg, raphe magnus nucleus; IRT, intermediate reticular nucleus; DCN, deep cerebellar nuclues.

After identifying the brain regions with monosynaptic input to LPT GABAergic neurons, the distribution of these DsRed-labeled afferent neurons was analyzed based on detailed statistics. We divided each brain into six general structures, namely the midbrain, diencephalon, telencephalon, cerebellum, medulla, and pons, which together encompassed 48 specific brain regions containing the DsRed-labeled neurons in the whole brain (*n* = 4 mice). Then we calculated the proportion of input from each region against the total number of input neurons (Figure 6, left). We found that the midbrain provided the highest numbers of inputs to the LPT, while few labeled neurons were found in the medulla or cerebellum. The largest number of inputs to LPT GABAergic neurons was found to arrive from the SC (15.10 ± 3.44%). The brain regions sending the second and third largest number of inputs to LPT GABAergic neurons were the SNr (10.00 ± 1.62%) and the DR (6.13 ± 1.07%) in the midbrain. In addition, the DpMe (5.17 ± 0.67%), PAG (3.64 ± 0.40%), and the substantia nigra pars compacta (SNc, 2.83 ± 0.68%) in the midbrain, the LHA (4.41 ± 1.05%) and PSTN (3.12 ± 1.08%) in the diencephalon, and the CeA (4.62 ± 1.71%) and the BNST (3.29 ± 0.48%) in the telencephalon also had strong projections to LPT GABAergic neurons.

### Output Patterns of LPT GABAergic Neurons

**FIGURE 7:**
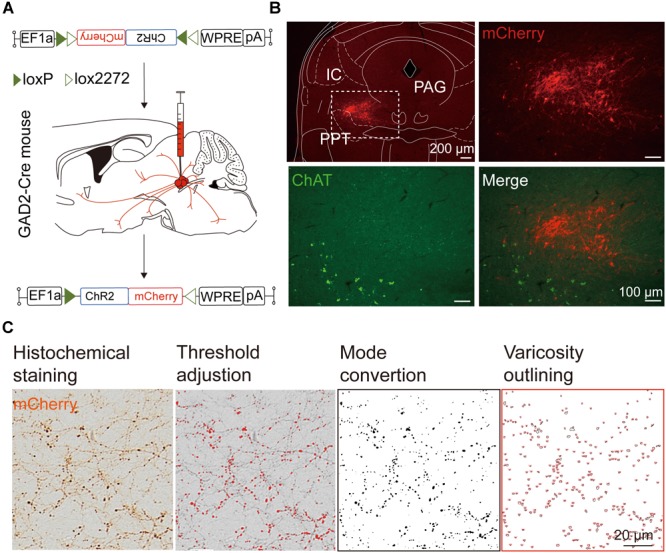
Experimental procedures and analysis of LPT GABAergic neuronal anterograde tracing. **(A)** A schematic of the viral vectors and injection site for AAV-EF1α-DIO-ChR2-mCherry in GAD2-IRES-Cre mice. **(B)** Fluorescence images showing that the virus injection was restricted to the LPT between the PPT (labeled in green by ChAT) and the PAG in a GAD2-IRES-Cre mouse. **(C)** Analysis of LPT GABAergic neuronal anterograde tracing. Left, typical axonal varicosities of LPT GABAergic neurons labeled by mCherry in the medial thalamus nucleus; Right, a sequence of three images showed the counting procedure of the mCherry-labeled axonal varicosities performed in ImageJ. PPT, pedunculopontine tegmental nucleus; PAG, periaqueductal gray; IC, inferior colliculus.

**FIGURE 8:**
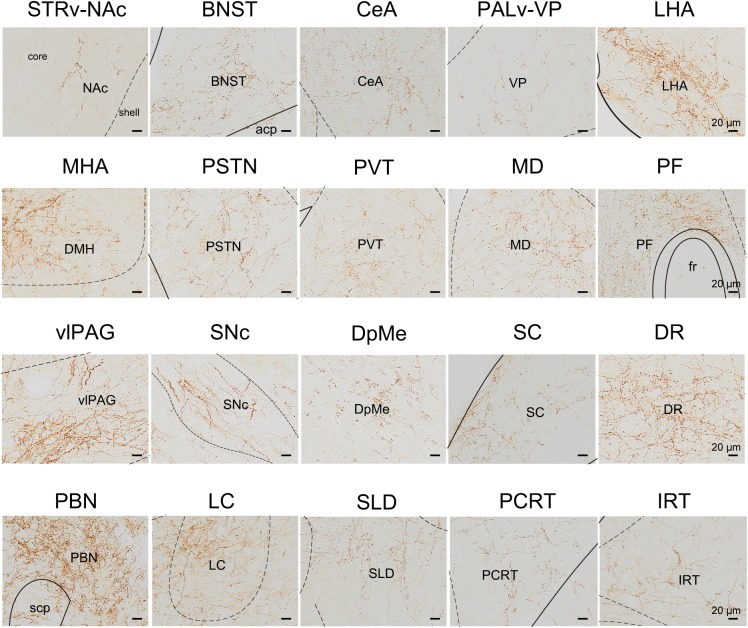
Schematic presenting the typical regions with LPT GABAergic neuronal outputs. These areas received LPT GABAergic innervation involved in sleep-wake regulation (e.g., LHA, LC, and SLD), locomotion (e.g., SC and SNc), emotion (e.g., CeA, and BNST), and reward control (e.g., VTA and NAc). Scale bar: 20 μm. STRv, stratum ventral region; NAc, nucleus accumbens; BNST, bed nucleus of the stria terminalis; CeA, central amygdaloid nucleus; PALv, pallidum, ventral region; VP, ventral pallidum; ZI, zona incerta; STN, subthalamic nucleus; PSTN, parasubthalamic nucleus; LHA, lateral hypothalamic area; MHA, medial hypothalamic area; PF, parafascicular thalamic nucleus; MD, mediodorsal thalamic nucleus; PVT, paraventricular thalamic nucleus; SNc, substantia nigra pars compacta; DpMe, deep mesencephalic nucleus; vlPAG, ventrolateral periaqueductal gray; SC, Superior colliculus; DR, dorsal raphe nucleus; PBN, parabrachial nucleus; SLD, sublateral dorsal nucleus; PCRt, parvicellular reticular nucleus, alpha part; IRT, intermediate reticular nucleus; LC, locus coeruleus; fr, fasciculus retroflexus; acp, anterior commissure, posterior; scp, superior cerebellar peduncle.

**FIGURE 9:**
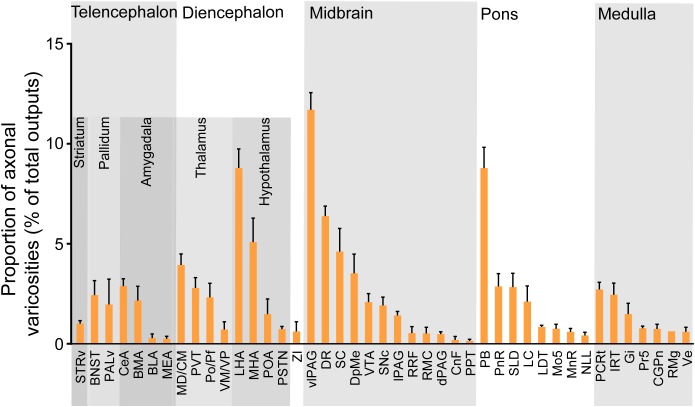
Statistical analysis of whole-brain outputs from LPT GABAergic neurons. Average ratio of axonal varicosity number in each brain region with no less than 0.1% average output proportions selected from four GAD2-IRES-Cre mice. Error bars represent the SEM. STRv, stratum ventral region; BNST, bed nucleus of the stria terminalis; PALv, pallidum, ventral region; CeA, central amygdaloid nucleus; MEA, medial amygdalar nucleus; BLA, basolateral amygdalar nucleus; BMA, basomedial amygdalar nucleus; MD/CM, mediodorsal/central media thalamic nucleus; PVT, paraventricular thalamic nucleus; PF/Po, parafascicular/posterior thalamic nucleus; VM/VP, ventromedial/ventroposterior thalamic nucleus; LHA, lateral hypothalamic area; MHA, medial hypothalamic area; PSTN, parasubthalamic nucleus; POA, preoptic area; ZI, zona incerta; SNc, substantia nigra pars compacta; SC, superior colliculus; DpMe, deep mesencephalic nucleus; VTA, ventral tegmental area; RRF, retrorubral field; RMC, red nucleus; CnF, cuneiform nucleus; PPT, pedunculopontine tegmental nucleus; DR, dorsal raphe nucleus; dPAG, dorsal periaqueductal gray; lPAG, lateral periaqueductal gray; vlPAG, ventrolateral periaqueductal gray; PnR, pontine reticular nuclues; LDT, laterodorsal tegmental nucleus; NLL, the nuclei of the lateral lemniscus; PBN, parabrachial nucleus; SLD, sublateral dorsal nucleus; LC, locus coeruleus; Mo5, motor trigeminal nucleus; MnR, median raphe nucleus; Gi, gigantocellular reticular nucleus; Ve, vestibular nucleus; PCRt, parvicellular reticular nucleus, alpha part; Pr5, principal sensory trigeminal nucleus; CGPn, central gray of the pons; RMg, raphe magnus nucleus; IRT, intermediate reticular nucleus.

We next mapped the output of LPT GABAergic neurons using an AAV-mediated virus, similar to our previous work with viral anterograde tracing (Zhang et al., 2013; Chen et al., 2016; Oishi et al., 2017; Wang et al., 2017). To label the axonal projections, we injected AAV expressing Cre-dependent ChR2-mCherry (Figure 7A) into the LPT of GAD2-IRES-Cre mice. After 3 weeks, mCherry expression was observed to be restricted to the LPT, between the PPT and vlPAG (Figure 7B). In order to more precisely detect axonal varicosities, we combined viral tracing with DAB immunostaining to label mCherry (Campos et al., 2014). Images were compared with the reference brain atlas, and mCherry labeled axons were detected (Figure 7C). After the neurons at the injection site (identified by the existence of mCherry labeled cell bodies) were excluded, the projection to each brain area was quantified by the pixels of the axonal varicosities (Figure 8). Among the five major brain subdivisions that received LPT GABAergic projections (the cerebellum received almost no projection), the midbrain, diencephalon, and pons received the most LPT GABAergic projections, while few axons were detected in the telencephalon or medulla (Figure 9, left). The largest proportion of projections from LPT GABAergic neurons was found in the vlPAG (11.70 ± 0.86%), whereas the proportion of the LPT GABAergic varicosities in other subregions of the PAG were 1.42 ± 0.20% (lPAG) and 0.49 ± 0.11% (dPAG). The DR (6.39 ± 0.50%) and SC (4.60 ± 1.16%) in the midbrain, the parabrachial nuclei (PBN, 8.79 ± 1.04%) in the pons, and the LHA (8.08 ± 0.94%), medial hypothalamic area (5.09 ± 1.20%), and mediodorsal/central medial thalamic nucleus (MD/CM, 3.94 ± 0.55%) in the diencephalon also received strong projections from LPT GABAergic neurons.

### Reciprocal Connections Between the LPT and Other Nuclei

**FIGURE 10:**
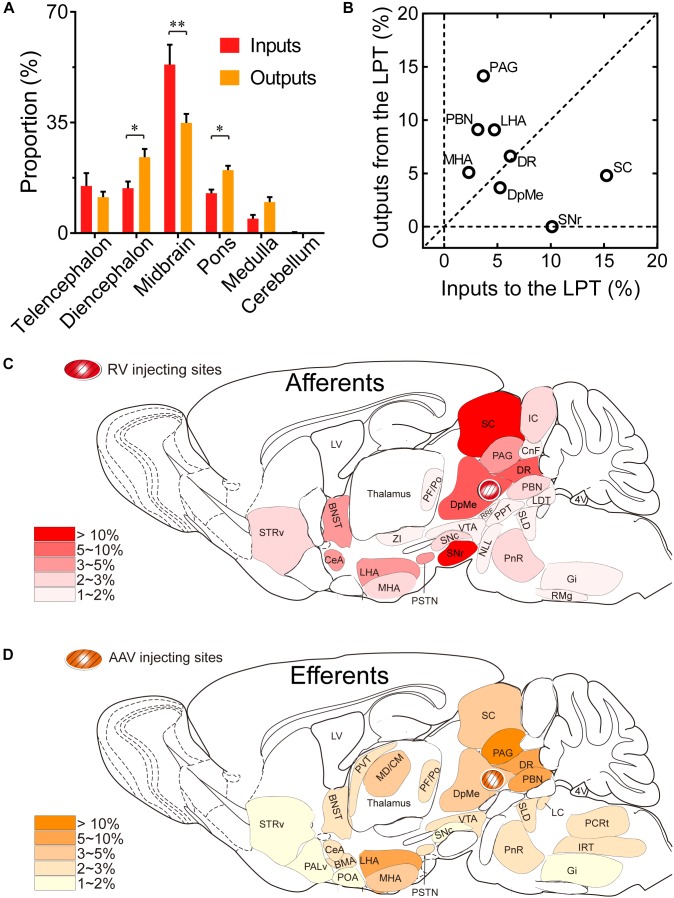
Comparison between inputs and outputs of LPT GABAergic neurons. **(A)** LPT GABAergic inputs and outputs were compared in six major structural subdivisions of the whole brain. LPT GABAergic inputs are colored red and the outputs are shaded in orange. The data represent the mean ± SEM, with n = 4 for each group. Two-tailed Student’s *t*-tests indicated significant (^∗^*P* < 0.05, ^∗∗^*P* < 0.01) differences between the inputs and outputs of LPT GABAergic neurons in the same subdivision. **(B)** The percentage of input versus percentage of output in each region of typical nuclei that had strong connections with the LPT. The proportion of inputs or outputs ≥ 5% are indicated with open circles. **(C,D)** Schematic diagrams presenting the afferents and efferents of LPT GABAergic neurons. BNST, bed nucleus of the stria terminalis; STRv, stratum ventral region; PALd, pallidum, dorsal region; PALv, pallidum, ventral region; CeA, central amygdaloid nucleus; BMA, basomedial amygdalar nucleus; POA, preoptic area; LHA, lateral hypothalamic area; MHA, medial hypothalamic area; PSTN, parasubthalamic nucleus; MD/CM, mediodorsal/central media thalamic nucleus; PVT, paraventricular thalamic nucleus; PF/Po, parafascicular/ posterior thalamic nucleus; SNc, substantia nigra pars compacta; ZI, zona incerta; SNr, substantia nigra pars reticulata; VTA, ventral tegmental area; DpMe, deep mesencephalic nucleus; SC, superior colliculus; RRF, retrorubral field; CnF, cuneiform nucleus; PPT, pedunculopontine tegmental nucleus; DR, dorsal raphe nucleus; PAG, periaqueductal gray; IC, inferior colliculus; PnR, pontine reticular nuclues; NLL, the nuclei of the lateral lemniscus; PBN, parabrachial nucleus; SLD, sublateral dorsal nucleus; LC, locus coeruleus; LDT, laterodorsal tegmental nucleus; IRT, intermediate reticular nucleus; Gi, gigantocellular reticular nucleus; PCRt, parvicellular reticular nucleus, alpha part.

Comparing the broad distribution of the inputs and outputs of the LPT, we found that there was a high correlation between LPT afferents and efferents on a large scale of the whole brain. We found the CC was high between the afferent and efferent spatial distributions in all six major brain subdivisions (*R* = 0.867). Among the six major brain subdivisions, the diencephalon, midbrain, and pons revealed strong reciprocal connections with the LPT; they heavily received projections from and sent axonal fibers to LPT GABAergic neurons. Specifically, the midbrain provided a significantly higher proportion of inputs to the LPT, compared with the proportion of projections from the LPT to the midbrain (53.31 ± 6.33 versus 34.87 ± 2.84%, *P* = 0.038; Individual *P*-values indicate two-tailed Student’s *t-*test comparisons of inputs and outputs for each brain region. In contrast, the pons and diencephalon had higher proportions of fibers arriving from the LPT than inputs going into the LPT (Figure 10A, pons: 19.94 ± 1.36 versus 12.62 ± 1.17%, *P* = 0.006; diencephalon: 24.03 ± 2.59 versus 14.22 ± 2.13%, *P* = 0.027 by two-tailed Student’s *t-*test).

Our analysis of the proportion of inputs versus outputs in each region revealed that LPT GABAergic neurons had equally interactive connections to structures within the DR in the midbrain (Figure 10B; the proportion of the inputs to the LPT versus the outputs from the LPT, 6.04 ± 1.07 versus 6.39 ± 0.50%). However, there were many brain regions where connections with the LPT dominated in a unidirectional manner, such as the PAG and SC (Figure 10B). Figure 10C,D summarizes the afferents and efferents of LPT GABAergic neurons in schematic form, reflecting the anatomical distribution of these innervations.

## Discussion

By applying advanced viral tracing methods, we identified for the first time the whole-brain distribution of input and output projections of LPT GABAergic neurons. The afferents and efferents of LPT GABAergic neurons were mostly asymmetric in major nuclei such as the LHA, SC, PAG, PBN, and SNr. Only the DR had equally reciprocal connections with the LPT. In addition, LPT GABAergic neurons received projections from several brain areas related to sleep-wake regulation and motor activity. Finally, we found a strong connection between LPT GABAergic neurons and the limbic system, suggesting that emotional processing may be associated with LPT-mediated REM sleep and motor regulation.

### Technical Advances and Considerations

Genetically engineered RV has been widely used in neuroscience for its cell type-specific infection of neurons that does not affect passing neuronal tracts and its effective passage across known synaptic connections (Wickersham et al., 2007; Weissbourd et al., 2014). Accordingly, our data obtained using RV-mediated retrograde tracing in GAD2-IRES-Cre mice demonstrated that there are, in fact, direct projections from the SLD and LHA to LPT GABAergic neurons. This result not only corroborated a similar finding using CTB retrograde tracing (Lu et al., 2006; Clement et al., 2012), but also firstly identified GABAergic neurons in the LPT that received the inputs from the SLD and LHA. In addition, there are some differences between the distributions of the inputs to LPT GABAergic neurons compared with the inputs to LPT non-specific neurons. Clement and colleagues report that the ZI sends numerus projections to the LPT by CTB tracing (Clement et al., 2012). However, we found that the specific connection from ZI to LPT GABAergic neurons by RV-based tracing was weak, suggesting that the ZI may send most projections to glutamatergic neurons in the LPT.

On the other side, we built a detailed and quantitative map of LPT GABAergic neuron projections to the entire brain by AAV-mediated anterograde tracing, which was more specific and more restricted than the traditional methods. For example, although the non-specific projections from the LPT to the subthalamic nucleus (STN) were found by biotinylated dextran amine (BDA) (Bevan and Bolam, 1995), they didn’t give the statistics of GABAergic projections from the LPT or full view of any other projecting nuclei. In our results, we quantitatively showed that LPT GABAergic neurons sent GABAergic varicosities in the STN (<0.1% of whole-brain varicosities). By visualizing the AAV reporter mCherry with DAB immunostaining, we avoided false positive signals, which are commonly observed after fluorescence immunohistochemistry (Oh et al., 2014). We chose to quantify projections by counting axonal varicosities instead of measuring fluorescence intensity, in order to avoid differences between thickness of brain sections or light exposure by the microscope.

### Morphological Findings

Due to the technical shortcomings of classical tracing methods, the complete neuronal connectivity of GABAergic neurons in the LPT was previously unknown. We used cell-type-specific anterograde and retrograde viral tracing to map the connectivity of LPT GABAergic neurons and found that their main inputs (≥20%) originated from the midbrain, whereas the brain regions that received projections from LPT GABAergic neurons were mainly distributed (≥20%) between the telencephalon, midbrain, and pons.

Among major nuclei connected to LPT GABAergic neurons, the DR had equally interactive connections to LPT GABAergic neurons (Figure 10B). Weissbourd and his colleagues found that the LPT projects to DR 5-HT neurons (Weissbourd et al., 2014). Here, we identified that the neuronal type in the LPT projected to the DR was GABAergic (Figure 8). The DR 5-HT neurons innervated LPT GABAergic neurons to form bidirectional connections (Figure 5F). This equally reciprocal LPT-DR connection may reveal a pathway for bidirectional feedback regulation of behavioral functions such as the antidepressant effect (Briley and Moret, 1993).

From comparison of distribution among the major nuclei more unidirectionally connecting to LPT GABAergic neurons, we found that afferents to LPT GABAergic neurons concentrated on the SC and SNr, whereas GABAergic efferents from the LPT were mostly distributed in the PAG, PBN, and LHA. Most afferents to LPT GABAergic neurons originated from the SC, particularly the intermediate and deep layers of the SC (Figure 3, 6). Although the projections from the SC to the LPT have been demonstrated in monkeys by anterograde tracing with BDA (Wang et al., 2013), the fact that these projections are predominantly GABAergic was unknown. Although previous studies indicated that the LPT received inhibitory projections from the SNr (Sherman et al., 2015), we identified the SNr as the second largest input to LPT GABAergic neurons. In addition, we showed that the main outputs of LPT GABAergic neurons project to the PAG, especially the vlPAG (Figure 8, 9), while LPT-projecting PAG neurons were already known (Weissbourd et al., 2014). It was previously suggested that the majority of LPT cells provide GABAergic inhibition of PPT cholinergic neurons, including those cholinergic neurons that provide a major ascending pathway into the posterior LHA (Ford et al., 1995). However, our data revealed that the LHA directly received strong projections from LPT GABAergic neurons.

The proportion of inputs to the LPT among whole-brain inputs is different from that of the LPT. A pathway from the PSTN to the LPT was previously reported using phaseolus vulgaris leucoagglutinin anterograde tracing (Goto and Swanson, 2004), and by this method it is not possible to determine whether a majority or a minority of PSTN neurons project to LPT. Here, we reported the proportion of the inputs from the PSTN projecting to LPT GABAergic neurons among whole-brain inputs was 3.02 ± 1.09%, which was about 44% colocalized with CR neurons (Figure 5C). Combined with dense inputs from the CeA in the forebrain, our results suggested a strong ability of the LPT to integrate behavioral information from the PSTN and CeA such as feeding and fear behaviors (Zimmerman et al., 2007; Chometton et al., 2016). Although the LPT has been shown to provide strong GABAergic projections to the dorsocaudal region of the pontine tegmentum, including the PBN, LC, and SLD (Hayashi et al., 2015), our finding of dense, LPT-originating, GABAergic axonal varicosities in the SNc of the midbrain suggested that the LPT also transmitted strong information to the SNc.

### Functional Implications

LPT GABAergic neurons have strong connections with brain regions related to sleep-wake regulation. Firstly, the output to the vlPAG is known to gate REM sleep and the ultradian rhythm of REM/NREM alternation (Weber et al., 2018). In addition, vlPAG/DpMe GABAergic neurons were shown to be important in sleep regulation (Hayashi et al., 2015; Weber and Dan, 2016) and thus, bidirectional projections between the LPT and vlPAG may be important for sleep regulation. Strong inhibitory projections from the LPT to the arousal-promoting PBN may also indicate a role of the LPT in sleep/wake control and thermoregulation (Fuller et al., 2011; Kaur et al., 2013; Nakamura, 2018; Wang et al., 2018). Furthermore, we and others identified SLD-projecting LPT GABAergic neurons (Boissard et al., 2003), which may play important roles in REM sleep regulation and related sleep disorders, such as narcolepsy or REM sleep behavior disorder. The direct connections we observed between the LPT and the hypothalamus suggested that LPT GABAergic neurons might be involved in regulation of hypothalamus functions, such as REM sleep, arousal, feeding behaviors, body temperature, and sense of pain (Clement et al., 2012; Li et al., 2014). Finally, another study showed that the neuronal activity of the posterior lateral hypothalamus containing the PSTN was related to the wake and REM sleep (Steininger et al., 1999). In the present study, the PSTN sent abundant projections to the LPT GABAergic neurons, suggesting that the pathway of the PSTN to LPT GABA neurons might be involved in sleep-wake regulation. Besides that, chemical activation of PSTN neurons elicited depressor as well as bradycardia (Ciriello et al., 2008), and hedonic tastes increased c-Fos expression of the PSTN in rats (Chometton et al., 2016), suggesting that cardiovascular regulation and food intake were probably mediated by the PSTN innervated LPT GABAergic neurons.

Anatomical connectivity of LPT GABAergic neurons with the SC, SN, and PAG in the midbrain may implicate a role of LPT in the regulation of motor behaviors. Our data revealed the existence of asymmetrically reciprocal circuits between the SC and LPT GABAergic neurons for the transmission of saccadic information. The LPT was suggested to be involved in the control of eye movements, due to a causal link that was reported between ocular flutter and small lesions in the right upper pontine tegmentum containing the LPT (Tsutsumi et al., 2009). Moreover, strong connections between the LPT and SN suggested a role in regulating locomotor behavior (Deng et al., 2016; Lintz and Felsen, 2016; Barchini et al., 2018; Weber et al., 2018). A study previously proposed that projections of SNr neurons to PPT glutamatergic neurons primarily regulated motor activity (Roseberry et al., 2016); however, the strong projections from the SNr to LPT GABAergic neurons may also implicate inhibitory LPT neurons in some aspects of motor control such as saccadic eye movement (Sato and Hikosaka, 2002). In contrast, SNc-projecting LPT GABAergic neurons were shown to be involved in motor and reward control (Pioli et al., 2008). Our finding that SNc neurons also modestly projected to LPT GABAergic neurons could indicate a sophisticated feedback mechanism between SNc and LPT GABAergic neurons.

Finally, the inputs from the limbic system, including the CeA and BNST, suggested that LPT GABAergic neurons may be regulated by neural circuits involved in stress and fear processing (Zhao and Davis, 2004). Cataplexy, a common symptom of narcolepsy, is known to be triggered by strong emotions associated with GABAergic neurons in the limbic system and may also result from the disturbance of REM sleep atonia (related to the LPT/vlPAG/LC) into wakefulness (Snow et al., 2017). Our data provided evidence that LPT GABAergic neurons directly received projections from BNST GABAergic neurons (Figure 4, 5D) and CeA GABAergic neurons (Figure 4, 5E). Therefore, we confirmed the anatomical connectivity of the limbic system with LPT GABAergic neurons that control REM sleep atonia, as hypothesized by Fuller (Peever and Fuller, 2017).

### Comparison With Areas Near the LPT

Although the vlPAG and PPT are in close proximity to the LPT, the connectivity of these GABAergic neurons is different from that of the LPT. Whereas vlPAG GABAergic neurons primarily receive inputs from the ventral medulla, the SLD, and the LHA (Boissard et al., 2003; Clement et al., 2012; Weber et al., 2015), the LPT is mainly innervated by the SC and SNr. In contrast to LPT GABAergic neurons, the vlPAG has major projections to the RMg and nearby areas, commonly known as the rostral ventral medulla (Bowman et al., 2013). Moreover, PPT GABAergic neurons are rarely innervated by the SNr (Roseberry et al., 2016), while LPT GABAergic neurons are strongly innervated by this area.

There are also clear differences in the physiological and pathological functions of the LPT, vlPAG, and PPT. GABAergic neurons in the vlPAG are important for the sleep-wake cycle, micturition, and nociception. In addition, optogenetic studies revealed that vlPAG GABAergic neurons regulated non-REM and REM sleep (Weber et al., 2018). Other studies based on genetically engineered systems showed that activation of vlPAG GABAergic neurons delayed detrusor contraction and inhibited voiding (Zare et al., 2018). In contrast, PPT GABAergic neurons are mostly involved in gait and balance regulation, a function that is not identified in LPT GABAergic neurons. For example, loss of rostral PPT GABAergic neurons was reported in Parkinson’s disease patients with gait and balance disorders (Sebille et al., 2018). In spite of the close proximity of the vlPAG, PPT, and LPT GABAergic neurons, it is likely that these areas have distinguishing physiological and pathological roles.

## Conclusion

We mapped, for the first time, the afferents and efferents of LPT GABAergic neurons and found them to be extensively interconnected with other brain areas. This suggested a vital role of LPT neurons in a wide range of physiological and pathological functions, especially sleep-wake regulation and locomotor control. Our anatomical data could be useful for future functional studies of the brain and also provide a structural basis to understand neurological disorders.

## Ethics Statement

This study was carried out in accordance with the recommendations of the China Regulations on the Administration of Laboratory Animals, the Decree No. 2 of National Science and Technology Commission of the People’s Republic of China and all animal procedures were approved by the Medical Experimental Animal Administrative Committee of the School of Basic Medical Sciences (Permit No. 20140226-024), Fudan University (Shanghai, China).

## Author Contributions

Z-KC, X-SY, and HD designed and performed the experiments, analyzed the data, and wrote the manuscript. Y-FW performed the experiments and analyzed the data. W-MQ and G-HC conceived the experiments and wrote the manuscript. MH provided GAD2-IRES-Cre mice. Z-LH conceived the experiments, analyzed the data, and wrote the manuscript.

## Conflict of Interest Statement

The authors declare that the research was conducted in the absence of any commercial or financial relationships that could be construed as a potential conflict of interest.

## References

[B1] Adamantidis A. R. Zhang F. Aravanis A. M. Deisseroth K. de Lecea L. (2007). Neural substrates of awakening probed with optogenetic control of hypocretin neurons. *Nature* 450 420–424. 10.1038/nature06310 17943086 PMC6744371

[B2] Alheid G. F. Milsom W. K. McCrimmon D. R. (2004). Pontine influences on breathing: an overview. *Respir. Physiol. Neurobiol.* 143 105–114. 10.1016/j.resp.2004.06.016 15519548

[B3] Appell P. P. Behan M. (1990). Sources of subcortical GABAergic projections to the superior colliculus in the cat. *J. Comp. Neurol.* 302 143–158. 10.1002/cne.903020111 2086611

[B4] Barchini J. Shi X. Chen H. Cang J. (2018). Bidirectional encoding of motion contrast in the mouse superior colliculus. *eLife* 7:e35261. 10.7554/eLife.35261 29963987 PMC6050041

[B5] Bevan M. D. Bolam J. P. (1995). Cholinergic, GABAergic, and glutamate-enriched inputs from the mesopontine tegmentum to the subthalamic nucleus in the rat. *J. Neurosci.* 15 7105–7120. 7472465 10.1523/JNEUROSCI.15-11-07105.1995 PMC6578076

[B6] Boissard R. Fort P. Gervasoni D. Barbagli B. Luppi P. H. (2003). Localization of the GABAergic and non-GABAergic neurons projecting to the sublaterodorsal nucleus and potentially gating paradoxical sleep onset. *Eur. J. Neurosci.* 18 1627–1639. 10.1046/j.1460-9568.2003.02861.x 14511341

[B7] Bowman B. R. Kumar N. N. Hassan S. F. McMullan S. Goodchild A. K. (2013). Brain sources of inhibitory input to the rat rostral ventrolateral medulla. *J. Comp. Neurol.* 521 213–232. 10.1002/cne.23175 22740031

[B8] Briley M. Moret C. (1993). Neurobiological mechanisms involved in antidepressant therapies. *Clin. Neuropharmacol.* 16 387–400. 8221701 10.1097/00002826-199310000-00002

[B9] Campos L. M. Cruz-Rizzolo R. J. Watanabe I. S. Pinato L. Nogueira M. I. (2014). Efferent projections of the suprachiasmatic nucleus based on the distribution of vasoactive intestinal peptide (VIP) and arginine vasopressin (AVP) immunoreactive fibers in the hypothalamus of *Sapajus apella*. *J. Chem. Neuroanat.* 5 42–53. 10.1016/j.jchemneu.2014.03.004 24727411

[B10] Chen L. Yin D. Wang T. X. Guo W. Dong H. Xu Q. (2016). Basal forebrain cholinergic neurons primarily contribute to inhibition of electroencephalogram delta activity, rather than inducing behavioral wakefulness in mice. *Neuropsychopharmacology* 41 2133–2146. 10.1038/npp.2016.13 26797244 PMC4908644

[B11] Chometton S. Pedron S. Peterschmitt Y. Van Waes V. Fellmann D. Risold P. Y. (2016). A premammillary lateral hypothalamic nuclear complex responds to hedonic but not aversive tastes in the male rat. *Brain Struct. Funct.* 221 2183–2208. 10.1007/s00429-015-1038-3 25863939

[B12] Ciriello J. Solano-Flores L. P. Rosas-Arellano M. P. Kirouac G. J. Babic T. (2008). Medullary pathways mediating the parasubthalamic nucleus depressor response. *Am. J. Physiol. Regul. Integr. Comp. Physiol.* 294 R1276–R1284. 10.1152/ajpregu.00437.2007 18287224

[B13] Clement O. Sapin E. Libourel P. A. Arthaud S. Brischoux F. Fort P. (2012). The lateral hypothalamic area controls paradoxical (REM) sleep by means of descending projections to brainstem GABAergic neurons. *J. Neurosci.* 32 16763–16774. 10.1523/JNEUROSCI.1885-12.2012 23175830 PMC6621764

[B14] Deng H. Xiao X. Wang Z. (2016). Periaqueductal gray neuronal activities underlie different aspects of defensive behaviors. *J. Neurosci.* 36 7580–7588. 10.1523/JNEUROSCI.4425-15.2016 27445137 PMC6705556

[B15] Englot D. J. Gonzalez H. F. J. Reynolds B. B. Konrad P. E. Jacobs M. L. Gore J. C. (2018). Relating structural and functional brainstem connectivity to disease measures in epilepsy. *Neurology* 91 e67–e77. 10.1212/WNL.0000000000005733 29848786 PMC6091881

[B16] Fogel S. M. Smith C. T. Beninger R. J. (2010). Increased GABAergic activity in the region of the pedunculopontine and deep mesencephalic reticular nuclei reduces REM sleep and impairs learning in rats. *Behav. Neurosci.* 124 79–86. 10.1037/a0018244 20141282

[B17] Ford B. Holmes C. J. Mainville L. Jones B. E. (1995). GABAergic neurons in the rat pontomesencephalic tegmentum: codistribution with cholinergic and other tegmental neurons projecting to the posterior lateral hypothalamus. *J. Comp. Neurol.* 363 177–196. 10.1002/cne.903630203 8642069

[B18] Franklin K. B. J. Paxinos G. (2001). *The Mouse Brain in Stereotaxic Coordinates: Compact* 2nd Edn. San Diego, CA: Academic Press.

[B19] Fuller P. M. Sherman D. Pedersen N. P. Saper C. B. Lu J. (2011). Reassessment of the structural basis of the ascending arousal system. *J. Comp. Neurol.* 519 933–956. 10.1002/cne.22559 21280045 PMC3119596

[B20] Gorbachevskaia A. I. (2011). [Interconnections of the pallidum, pedunculopontine nucleus, zona incerta, and deep mesencephalic nucleus–the structures of the basal ganglia morpho-functional system]. *Morfologiia* 139 19–24. 21954703

[B21] Goto M. Swanson L. W. (2004). Axonal projections from the parasubthalamic nucleus. *J. Comp. Neurol.* 469 581–607. 10.1002/cne.11036 14755537

[B22] Hayashi Y. Kashiwagi M. Yasuda K. Ando R. Kanuka M. Sakai K. (2015). Cells of a common developmental origin regulate REM/non-REM sleep and wakefulness in mice. *Science* 350 957–961. 10.1126/science.aad1023 26494173

[B23] Holloway B. B. Stornetta R. L. Bochorishvili G. Erisir A. Viar K. E. Guyenet P. G. (2013). Monosynaptic glutamatergic activation of locus coeruleus and other lower brainstem noradrenergic neurons by the C1 cells in mice. *J. Neurosci.* 33 18792–18805. 10.1523/JNEUROSCI.2916-13.2013 24285886 PMC3841449

[B24] Ito H. Yanase M. Yamashita A. Kitabatake C. Hamada A. Suhara Y. (2013). Analysis of sleep disorders under pain using an optogenetic tool: possible involvement of the activation of dorsal raphe nucleus-serotonergic neurons. *Mol. Brain* 6:59. 10.1186/1756-6606-6-59 24370235 PMC3879646

[B25] Kaur S. Pedersen N. P. Yokota S. Hur E. E. Fuller P. M. Lazarus M. (2013). Glutamatergic signaling from the parabrachial nucleus plays a critical role in hypercapnic arousal. *J. Neurosci.* 33 7627–7640. 10.1523/JNEUROSCI.0173-13.2013 23637157 PMC3674488

[B26] Kodani S. Soya S. Sakurai T. (2017). Excitation of GABAergic neurons in the bed nucleus of the stria terminalis triggers immediate transition from non-rapid eye movement sleep to wakefulness in mice. *J. Neurosci.* 37 7164–7176. 10.1523/JNEUROSCI.0245-17.2017 28642284 PMC6705739

[B27] Krenzer M. Anaclet C. Vetrivelan R. Wang N. Vong L. Lowell B. B. (2011). Brainstem and spinal cord circuitry regulating REM sleep and muscle atonia. *PLoS One* 6:e24998. 10.1371/journal.pone.0024998 22043278 PMC3197189

[B28] Kuhlman S. J. Huang Z. J. (2008). High-resolution labeling and functional manipulation of specific neuron types in mouse brain by Cre-activated viral gene expression. *PLoS One* 3:e2005. 10.1371/journal.pone.0002005 18414675 PMC2289876

[B29] Le Ray D. Juvin L. Ryczko D. Dubuc R. (2011). Chapter 4–supraspinal control of locomotion: the mesencephalic locomotor region. *Prog. Brain Res.* 188 51–70. 10.1016/B978-0-444-53825-3.00009-7 21333802

[B30] Li J. Hu Z. de Lecea L. (2014). The hypocretins/orexins: integrators of multiple physiological functions. *Br. J. Pharmacol.* 171 332–350. 10.1111/bph.12415 24102345 PMC3904255

[B31] Li R. Nishijo H. Ono T. Ohtani Y. Ohtani O. (2002). Synapses on GABAergic neurons in the basolateral nucleus of the rat amygdala: double-labeling immunoelectron microscopy. *Synapse* 43 42–50. 10.1002/syn.10017 11746732

[B32] Liang C. L. Marks G. A. (2014). GABAA receptors are located in cholinergic terminals in the nucleus pontis oralis of the rat: implications for REM sleep control. *Brain Res.* 1543 58–64. 10.1016/j.brainres.2013.10.019 24141149

[B33] Liang C. L. Quang Nguyen T. Marks G. A. (2014). Inhibitory and excitatory amino acid neurotransmitters are utilized by the projection from the dorsal deep mesencephalic nucleus to the sublaterodorsal nucleus REM sleep induction zone. *Brain Res.* 1567 1–12. 10.1016/j.brainres.2014.04.016 24751569 PMC4077466

[B34] Lintz M. J. Felsen G. (2016). Basal ganglia output reflects internally-specified movements. *eLife* 5:e13833. 10.7554/eLife.13833 27377356 PMC4970866

[B35] Lu J. Sherman D. Devor M. Saper C. B. (2006). A putative flip-flop switch for control of REM sleep. *Nature* 441 589–594. 10.1038/nature04767 16688184

[B36] Luo Y. J. Li Y. D. Wang L. Yang S. R. Yuan X. S. Wang J. (2018). Nucleus accumbens controls wakefulness by a subpopulation of neurons expressing dopamine D1 receptors. *Nat. Commun.* 9:1576. 10.1038/s41467-018-03889-3 29679009 PMC5910424

[B37] Mahoney C. E. Agostinelli L. J. Brooks J. N. Lowell B. B. Scammell T. E. (2017). GABAergic neurons of the central amygdala promote cataplexy. *J. Neurosci.* 37 3995–4006. 10.1523/JNEUROSCI.4065-15.2017 28235898 PMC5391681

[B38] Mechawar N. Cozzari C. Descarries L. (2000). Cholinergic innervation in adult rat cerebral cortex: a quantitative immunocytochemical description. *J. Comp. Neurol.* 428 305–318. 11064369 10.1002/1096-9861(20001211)428:2<305::aid-cne9>3.0.co;2-y

[B39] Nakamura K. (2018). [Thermoregulatory behavior and its central circuit mechanism-What thermosensory pathway drives it?]. *Clin. Calcium* 28 65–72. 29279428

[B40] Oh S. W. Harris J. A. Ng L. Winslow B. Cain N. Mihalas S. (2014). A mesoscale connectome of the mouse brain. *Nature* 508 207–214. 10.1038/nature13186 24695228 PMC5102064

[B41] Oishi Y. Xu Q. Wang L. Zhang B. J. Takahashi K. Takata Y. (2017). Slow-wave sleep is controlled by a subset of nucleus accumbens core neurons in mice. *Nat. Commun.* 8:734. 10.1038/s41467-017-00781-4 28963505 PMC5622037

[B42] Pal D. Mallick B. N. (2009). GABA in pedunculopontine tegmentum increases rapid eye movement sleep in freely moving rats: possible role of GABA-ergic inputs from substantia nigra pars reticulata. *Neuroscience* 164 404–414. 10.1016/j.neuroscience.2009.08.025 19698764

[B43] Peever J. Fuller P. M. (2017). The Biology of REM Sleep. *Curr. Biol.* 27 R1237–R1248. 10.1016/j.cub.2017.10.026 29161567

[B44] Pioli E. Y. Meissner W. Sohr R. Gross C. E. Bezard E. Bioulac B. H. (2008). Differential behavioral effects of partial bilateral lesions of ventral tegmental area or substantia nigra pars compacta in rats. *Neuroscience* 153 1213–1224. 10.1016/j.neuroscience.2008.01.084 18455318

[B45] Plazzi G. Montagna P. Provini F. Bizzi A. Cohen M. Lugaresi E. (1996). Pontine lesions in idiopathic narcolepsy. *Neurology* 46 1250–1254. 10.1212/WNL.46.5.1250 8628461

[B46] Roseberry T. K. Lee A. M. Lalive A. L. Wilbrecht L. Bonci A. Kreitzer A. C. (2016). Cell-type-specific control of brainstem locomotor circuits by basal ganglia. *Cell* 164 526–537. 10.1016/j.cell.2015.12.037 26824660 PMC4733247

[B47] Sapin E. Lapray D. Berod A. Goutagny R. Leger L. Ravassard P. (2009). Localization of the brainstem GABAergic neurons controlling paradoxical (REM) sleep. *PLoS One* 4:e4272. 10.1371/journal.pone.0004272 19169414 PMC2629845

[B48] Sasaki K. Suzuki M. Mieda M. Tsujino N. Roth B. Sakurai T. (2011). Pharmacogenetic modulation of orexin neurons alters sleep/wakefulness states in mice. *PLoS One* 6:e20360. 10.1371/journal.pone.0020360 21647372 PMC3103553

[B49] Sato M. Hikosaka O. (2002). Role of primate substantia nigra pars reticulata in reward-oriented saccadic eye movement. *J. Neurosci.* 22 2363–2373. 10.1523/JNEUROSCI.22-06-02363.2002 11896175 PMC6758246

[B50] Scherfler C. Frauscher B. Schocke M. Iranzo A. Gschliesser V. Seppi K. (2011). White and gray matter abnormalities in idiopathic rapid eye movement sleep behavior disorder: a diffusion-tensor imaging and voxel-based morphometry study. *Ann. Neurol.* 69 400–407. 10.1002/ana.22245 21387382

[B51] Sebille S. B. Rolland A. S. Faillot M. Perez-Garcia F. Colomb-Clerc A. Lau B. (2018). Normal and pathological neuronal distribution of the human mesencephalic locomotor region. *Mov. Disord.* 34 218–227. 10.1002/mds.27578 30485555

[B52] Sherman D. Fuller P. M. Marcus J. Yu J. Zhang P. Chamberlin N. L. (2015). Anatomical location of the mesencephalic locomotor region and its possible role in locomotion, posture, cataplexy, and Parkinsonism. *Front. Neurol.* 6:140. 10.3389/fneur.2015.00140 26157418 PMC4478394

[B53] Snow M. B. Fraigne J. J. Thibault-Messier G. Chuen V. L. Thomasian A. Horner R. L. (2017). GABA cells in the central nucleus of the amygdala promote cataplexy. *J. Neurosci.* 37 4007–4022. 10.1523/JNEUROSCI.4070-15.2017 28209737 PMC6596591

[B54] Steininger T. L. Alam M. N. Gong H. Szymusiak R. McGinty D. (1999). Sleep-waking discharge of neurons in the posterior lateral hypothalamus of the albino rat. *Brain Res.* 840 138–147. 10.1016/S0006-8993(99)01648-0 10517961

[B55] Sukhotinsky I. Hopkins D. A. Lu J. Saper C. B. Devor M. (2005). Movement suppression during anesthesia: neural projections from the mesopontine tegmentum to areas involved in motor control. *J. Comp. Neurol.* 489 425–448. 10.1002/cne.20636 16025457

[B56] Taniguchi H. He M. Wu P. Kim S. Paik R. Sugino K. (2011). A resource of Cre driver lines for genetic targeting of GABAergic neurons in cerebral cortex. *Neuron* 71 995–1013. 10.1016/j.neuron.2011.07.026 21943598 PMC3779648

[B57] Tsutsumi T. Murakami M. Kawaishi J. Chida W. Watanabe K. (2009). Ocular flutter associated with a lesion of the right upper pontine tegmentum. *Auris Nasus Larynx* 36 695–697. 10.1016/j.anl.2009.02.007 19410400

[B58] Tziridis K. Dicke P. W. Thier P. (2012). Pontine reference frames for the sensory guidance of movement. *Cereb. Cortex* 22 345–362. 10.1093/cercor/bhr109 21670098

[B59] Vetrivelan R. Kong D. Ferrari L. L. Arrigoni E. Madara J. C. Bandaru S. S. (2016). Melanin-concentrating hormone neurons specifically promote rapid eye movement sleep in mice. *Neuroscience* 336 102–113. 10.1016/j.neuroscience.2016.08.046 27595887 PMC5056843

[B60] Vita M. G. Batocchi A. P. Dittoni S. Losurdo A. Cianfoni A. Stefanini M. C. (2008). Visual hallucinations and pontine demyelination in a child: possible REM dissociation? *J. Clin. Sleep Med.* 4 588–590. 19110890 PMC2603538

[B61] Wang N. Perkins E. Zhou L. Warren S. May P. J. (2013). Anatomical evidence that the superior colliculus controls saccades through central mesencephalic reticular formation gating of omnipause neuron activity. *J. Neurosci.* 33 16285–16296. 10.1523/JNEUROSCI.2726-11.2013 24107960 PMC3792464

[B62] Wang T. X. Xiong B. Xu W. Wei H. H. Qu W. M. Hong Z. Y. (2018). Activation of parabrachial nucleus glutamatergic neurons accelerates reanimation from sevoflurane anesthesia in mice. *Anesthesiology* 130 106–118. 10.1097/ALN.0000000000002475 30325744

[B63] Wang Y. Q. Li R. Wang D. R. Cherasse Y. Zhang Z. Zhang M. Q. (2017). Adenosine A2A receptors in the olfactory bulb suppress rapid eye movement sleep in rodents. *Brain Struct. Funct.* 222 1351–1366. 10.1007/s00429-016-1281-2 27485749

[B64] Weber F. Chung S. Beier K. T. Xu M. Luo L. Dan Y. (2015). Control of REM sleep by ventral medulla GABAergic neurons. *Nature* 526 435–438. 10.1038/nature14979 26444238 PMC4852286

[B65] Weber F. Dan Y. (2016). Circuit-based interrogation of sleep control. *Nature* 538 51–59. 10.1038/nature19773 27708309

[B66] Weber F. Hoang Do J. P. Chung S. Beier K. T. Bikov M. Saffari Doost M. (2018). Regulation of REM and Non-REM Sleep by Periaqueductal GABAergic Neurons. *Nat. Commun.* 9:354. 10.1038/s41467-017-02765-w 29367602 PMC5783937

[B67] Weissbourd B. Ren J. DeLoach K. E. Guenthner C. J. Miyamichi K. Luo L. (2014). Presynaptic partners of dorsal raphe serotonergic and GABAergic neurons. *Neuron* 83 645–662. 10.1016/j.neuron.2014.06.024 25102560 PMC4779447

[B68] Wickersham I. R. Lyon D. C. Barnard R. J. Mori T. Finke S. Conzelmann K. K. (2007). Monosynaptic restriction of transsynaptic tracing from single, genetically targeted neurons. *Neuron* 53 639–647. 10.1016/j.neuron.2007.01.033 17329205 PMC2629495

[B69] Yuan X. S. Wang L. Dong H. Qu W. M. Yang S. R. Cherasse Y. (2017). Striatal adenosine A2A receptor neurons control active-period sleep via parvalbumin neurons in external globus pallidus. *eLife* 6:e29055. 10.7554/eLife.29055 29022877 PMC5655138

[B70] Yuan X. S. Wei H. H. Xu W. Wang L. Qu W. M. Li R. X. (2018). Whole-brain monosynaptic afferent projections to the cholecystokinin neurons of the suprachiasmatic. *Front. Neurosci.* 12:807. 10.3389/fnins.2018.00807 30455627 PMC6230653

[B71] Zare A. Jahanshahi A. Rahnama’i M. S. Schipper S. van Koeveringe G. A. (2018). The role of the periaqueductal gray matter in lower urinary tract function. *Mol. Neurobiol.* 56 920–934. 10.1007/s12035-018-1131-8 29804231 PMC6400878

[B72] Zhang J. P. Xu Q. Yuan X. S. Cherasse Y. Schiffmann S. N. de Kerchove d’Exaerde A. (2013). Projections of nucleus accumbens adenosine A2A receptor neurons in the mouse brain and their implications in mediating sleep-wake regulation. *Front. Neuroanat.* 7:43. 10.3389/fnana.2013.00043 24409122 PMC3857888

[B73] Zhang Z. Wang H. J. Wang D. R. Qu W. M. Huang Z. L. (2017). Red light at intensities above 10 lx alters sleep-wake behavior in mice. *Light Sci. Appl.* 6:e16231. 10.1038/lsa.2016.231 30167247 PMC6062196

[B74] Zhao Z. Davis M. (2004). Fear-potentiated startle in rats is mediated by neurons in the deep layers of the superior colliculus/deep mesencephalic nucleus of the rostral midbrain through the glutamate non-NMDA receptors. *J. Neurosci.* 24 10326–10334. 10.1523/JNEUROSCI.2758-04.2004 15548646 PMC6730294

[B75] Zimmerman J. M. Rabinak C. A. McLachlan I. G. Maren S. (2007). The central nucleus of the amygdala is essential for acquiring and expressing conditional fear after overtraining. *Learn. Mem.* 14 634–644. 10.1101/lm.607207 17848503 PMC1994080

